# Adult Radiographic Presentation of Corpus Callosal Agenesis With a Single Interhemispheric Cyst and Dandy-Walker Malformation: A Case Report

**DOI:** 10.7759/cureus.38396

**Published:** 2023-05-01

**Authors:** Tyler Morgan, John Ciubuc, Dylan Murray, Matthew Murray, Richard Murray

**Affiliations:** 1 Surgery, Texas Tech University Health Sciences Center, Amarillo, USA; 2 Surgery, University College Dublin, Dublin, IRL; 3 Surgery, Royal College of Surgeons in Ireland, Dublin, IRL; 4 Diagnostic Radiology, Texas Tech University Health Sciences Center, Amarillo, USA

**Keywords:** type 1a cyst, barkovich, monoventricle, embryology, holoprosencephaly, interhemispheric cyst, dandy-walker, corpus callosum agenesis, neuroradiology

## Abstract

Agenesis or dysgenesis of the corpus callosum may occur due to ischemic, toxic, traumatic, or another insult to the fetus in the first trimester. Occasionally, such a malformation is associated with an interhemispheric cyst, among other central nervous system anomalies. Holoprosencephaly tends to mimic this radiographic presentation, which is where key imaging findings are helpful to differentiate between these entities. We present a 56-year-old male patient who was found to have a monoventricle, corpus callosum agenesis, interhemispheric cyst, and a Dandy-Walker malformation. The patient presented with a right acetabular fracture with computed tomography (CT) of the brain revealing the congenital brain abnormalities. The patient’s past medical history was notable for a seizure disorder identified during early adulthood. The CT scan of the head revealed a large monoventricle with an associated midline dorsal interhemispheric cyst and a Dandy-Walker malformation. The absence of both the corpus callosum and septum pellucidum was noted, with the presence of a monoventricle, leading to an initial differential of holoprosencephaly. Further review of the findings suggested instead a rare congenital presentation consisting of corpus callosum agenesis and an interhemispheric cyst. This case highlights a unique radiographic presentation of multiple brain anomalies, rarely presented in non-pediatric literature, which may help determine appropriate surgical and medical management for similarly affected adult individuals.

## Introduction

Structural brain abnormalities occur in approximately one to two in every 1000 births [1]. The causes can vary and include genetic disorders, both sporadic and inherited, congenitally acquired infections including toxoplasmosis, rubella, cytomegalovirus, herpes virus, as well as other fetal insults. The development of the corpus callosum happens between the twelfth (12) and twentieth (20) week of pregnancy and progresses from the front (anterior body and genu) to the back (splenium) [2]. If there is an insult during the eighth (8) to twelfth (12) week of gestation, it may result in corpus callosum agenesis (CCA), which is a common manifestation among central nervous system malformations [3]. CCA is rarely isolated and is often accompanied by other central nervous system abnormalities, such as Dandy-Walker malformation, holoprosencephaly, cerebellar and vermian hypoplasia, sulcal abnormalities, and ventriculomegaly [1, 2]. Interhemispheric cysts, which are less common in this context, occur in about 7% of patients with CCA and can be classified into two groups: type 1, which communicates with the ventricular system, and type 2, which does not [4, 5].

The prognosis of individuals with CCA is favorable, with limited impact on general functional capacity. Delays in development, including difficulties in abstract thinking and problem-solving, are often observed [6]. The symptoms of the central nervous system can range from normal to epilepsy and motor issues, depending on associated conditions such as interhemispheric cysts and Dandy-Walker malformations [5]. Non-central nervous system anomalies primarily involve the cranial-facial region, manifesting as macrocephaly, hypertelorism, nasal bridge malformations, and cleft lip and/or palate. These anomalies are thought to stem from the same embryonic defect as CCA due to their shared midline abnormalities [7]. The co-presentation of CCA, interhemispheric cyst, and Dandy-Walker malformation have been previously reported, but have been primarily focused on the pediatric population; rarely being reported in adult case report studies.

Imaging results are crucial in determining the cause of a patient's symptoms, particularly those presenting with cerebral abnormalities. Among those presenting with a monoventricle, especially with accompanying midline cranial defects, holoprosencephaly is a dominant differential to consider. The criteria defining holoprosencephaly focuses on the fusion of midline central nervous system structures which include the frontal lobes, basal ganglia, and thalamic bodies [8]. In the absence of fused structures, an alternative diagnosis should be considered.

## Case presentation

A 56-year-old male was admitted to the hospital with a history of trauma. The height and weight of the patient were 172.7 cm and 95.5 kg respectively. He had a history of chronic hypertension with elevated serum cholesterol. Table 1 shows the patient's vital parameters at the time of admission.

**Table 1:** Vital signs on admission

Vital signs on admission		
Vital signs	Patient measurements	Normal range
Heart rate	95 beats/minute	60-100 beats/minute
Respiratory rate	25 times/minute	12-20 times/minute
Systolic blood pressure	172 mmHg	90-120 mmHg
Diastolic blood pressure	78 mmHg	50-80 mmHg
SpO2 (room air)	99%	95–100%
Height	172.7 cm	Varies
Weight	95.5 kg	Varies

He was diagnosed with Dandy-Walker malformation with associated hydrocephalus as a child and subsequently developed a seizure disorder at age nineteen. Prior to this age, the patient's development was not unusual as per his mother aside from occasional hyponatremia events that manifested as fainting spells. He has mild cognitive deficits and requires some assistance with activities of daily living. The patient has a history of multiple episodes of urinary retention that required urologic consultation. He does not work and receives disability due to a seizure disability controlled with citalopram and phenobarbital. The patient's family history is noncontributory, and additional childhood medical history is not available.

**Figure 1:**
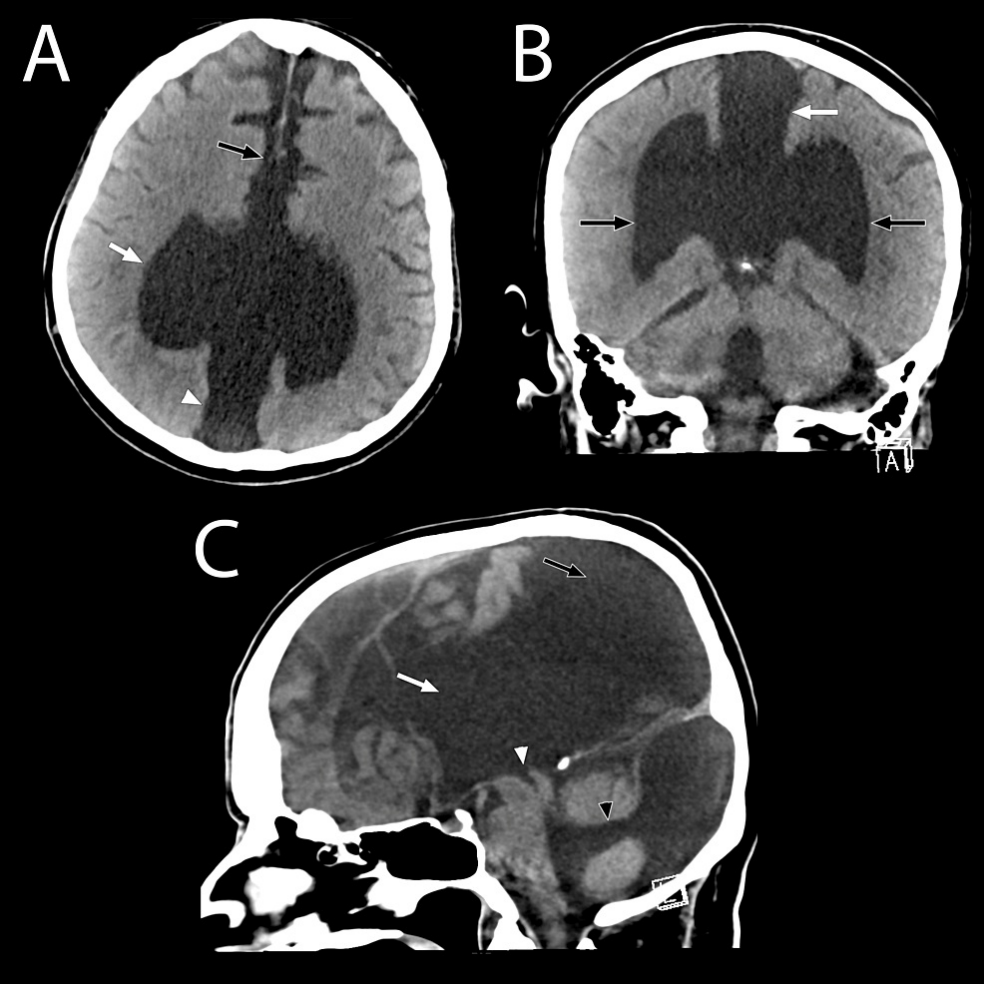
(A) Axial CT of the brain; (B) Coronal CT of the brain; (C) Midline Sagittal CT of the brain at the level of the medial longitudinal fissure. (A) The dorsal, midline, interhemispheric cyst (white arrowhead) is demonstrated along with its contiguous connection with the monoventricle (white arrow). A key feature identified in this view is the visibly separated frontal lobes (black arrow), with the presence of the falx cerebri, displaying non-fusion in the presence of an absent septum pellucidum. (B) The midline dorsal interhemispheric cyst (white arrow) communicates directly with the monoventricle. In this view, the monoventricle, which includes the third ventricle and the two lateral ventricles (indicated by black arrows), is clearly discernible. Note the clearly demarcated open communication between the dorsal interhemispheric cyst and the monoventricle, along with the absence of the corpus callosum. (C) The large monoventricle (white arrow) communicates with the dorsal interhemispheric cyst (black arrow) and is well appreciated. The Sylvian aqueduct draining the third ventricular component of the monoventricle is identified with the white arrowhead. The communication between the fourth ventricle (black arrowhead) and the posterior fossa cyst is evident in this view, in addition to the posterior fossa cyst. This view well demonstrates the essential components of the Dandy-Walker malformation and notes the absence of the corpus callosum.

**Figure 2:**
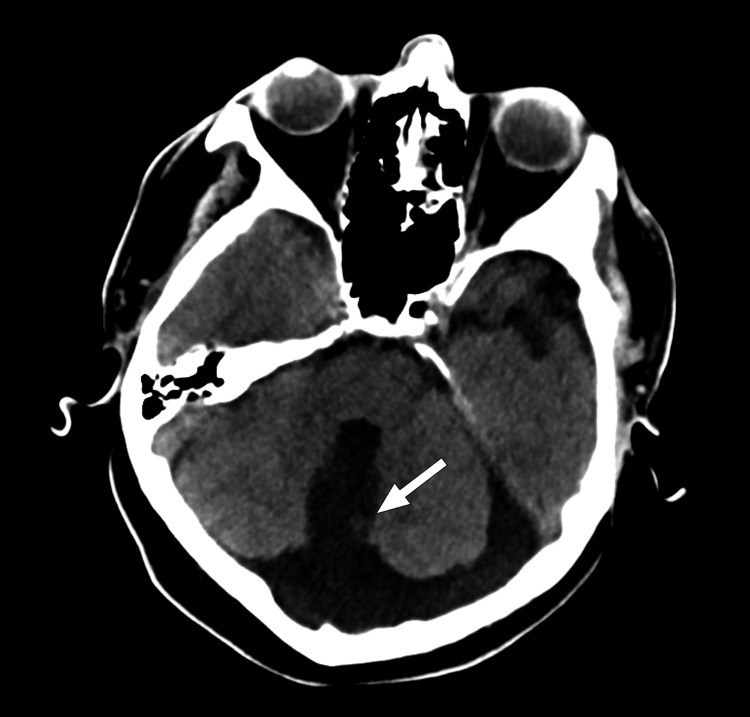
Axial CT of the brain at the level of the fourth ventricle. The Dandy-Walker malformation comprising the fourth ventricle connecting (white arrow) with the posterior fossa cyst is visualized in this view.

On examination, the muscle strength in the upper and left lower extremity was normal, but while examining the right lower limb, the patient experience pain. X-Ray imaging of the patient's pelvis revealed a comminuted fracture involving the right acetabulum. There was occasional choreiform-like movement involving the hands and arms. A computed tomography scan of the head taken at this time showed several congenital malformations of the brain including the presence of a monoventricle composed of the lateral ventricles and third ventricle, with a contiguous dorsal interhemispheric cyst arising from the monoventricle postero-superiorly. In addition, the absence of the septum pellucidum and callosal agenesis were present. In the posterior fossa, separate from the aforementioned findings, a Dandy-Walker malformation, i.e. a posterior fossa cyst communicating with the fourth ventricle, was present (Figures 1, 2). There was no identified facial malformation.

## Discussion

The isolated absence of the corpus callosum is often without symptoms, making it difficult to accurately estimate its frequency. Autopsy results indicate that CCA may occur as rarely as 2.5 per 10,000 births [9]. Clinically, however, it may be over-represented, as it is frequently associated with other abnormalities that cause symptoms. Two such associated anomalies are interhemispheric cysts and Dandy-Walker malformations, which the latter is seen in around 10% of these patients [10].

In 2001, Barkovich et al. developed a classification system for the various types of cysts that may occur in conjunction with agenesis of the corpus callosum [11]. The system was based on the analysis of patients whose imaging showed an interhemispheric cyst and corpus callosum anomalies. They divided the cysts into two main categories: type 1 and type 2 cysts. Type 1 cysts communicated with the ventricles, while type 2 cysts appeared as isolated structures that did not communicate with the ventricles. Type 1 cysts were further subdivided into subtypes 1a, 1b, and 1c, and type 2 cysts were divided into subtypes 2a, 2b, 2c, and 2d. Type 1a cysts were linked to communicating hydrocephalus with no other apparent brain malformations. Hydrocephalus may be associated with compression of brain tissue and may lead to some difficulties in brain function and even mental retardation if not treated [12]. Type 1b cysts were associated with obstructive hydrocephalus due to blockage of cerebrospinal fluid outflow from the third ventricle. Type 1c cysts were associated with cerebral hypoplasia and small skull size. Elucidating this correlation, the development of the bones of the skull is closely related to the development of the brain beneath. Any developmental abnormality in the brain, such as failure of the neural tube to close, can present as anencephaly [13]. Type 2a cysts were related to callosal agenesis or hypogenesis but without any other abnormalities. Type 2b cysts were multi-loculated and associated with deficiencies in the falx cerebri, polymicrogyria, and subependymal heterotopia. Type 2c cysts were linked to subcortical heterotopia. Type 2d cysts were defined as arachnoid cysts and were not seen in any of the patients studied, but are well documented in the literature.

The present case aligns with the 1a subtype according to Barkovich et al.'s classification system [11]. The study found that all patients assigned to the 1a subtype were male and developed hydrocephalus early in life, similar to the patient described in this paper. Barkovich et al. speculated that the 1a subtype may be a form of X-linked hydrocephalus, but this hypothesis has not been confirmed through genetic testing.

The treatment approach for individuals with interhemispheric cysts and agenesis of the corpus callosum is still a topic of debate. Possible treatments include cyst fenestration through craniotomy, neuroendoscopic fenestration, or ventricular shunt placement [12]. Currently, there is a discussion if early intervention in asymptomatic individuals results in improved outcomes. Recent discussions focused on monitoring asymptomatic individuals closely for the emergence of symptoms before intervening [14]. Symptoms due to increased cranial pressure, such as headaches, dizziness, epilepsy, and psychomotor retardation, are the main indicators for intervention. However, the relationship between interhemispheric cysts and increased cranial pressure is not clearly established [14]. As such, surgical intervention to resect an interhemispheric cyst, which may not be the causal entity, can result in suboptimal outcomes in as much as 72% of patients [15]. In comparison, in patients where the cyst is the likely causal entity, intervention can lead to substantially improved outcomes through the reduction of cranial pressure [14, 15].

**Figure 3:**
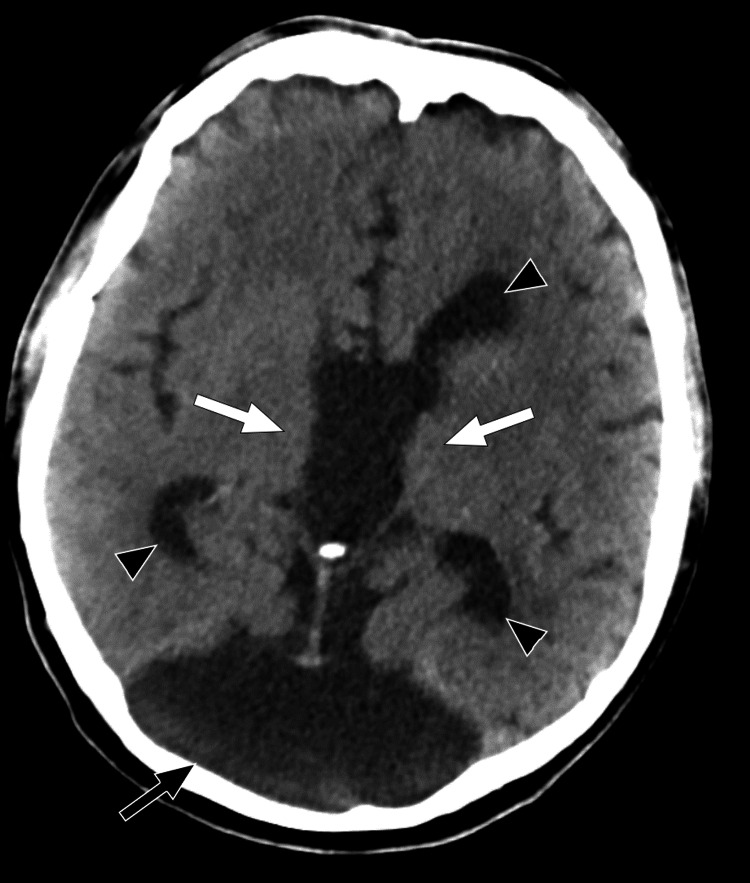
Axial CT of the brain at the level of the thalamus reveals an absence of thalamic fusion (white arrows). The third ventricular component of the monoventricle, along with the frontal horns and trigone of the lateral ventricular components (black arrowheads), can be seen at this level. Additionally, the posterior fossa cyst of the Dandy-Walker malformation (black arrow) is partially visible.

**Figure 4:**
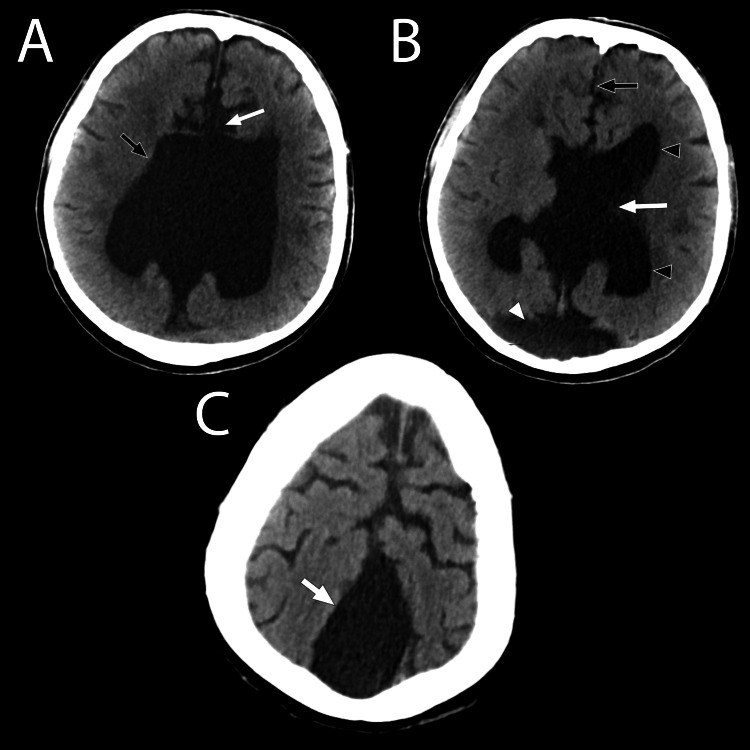
Axial CT series of the brain at various levels A, B, and C. (A) The monoventricle (black arrow) and absence of the corpus callosum (white arrow) are appreciated in this view. (B) The monoventricle (white arrow) and the posterior fossa cyst (white arrowhead) of the Dandy-Walker malformation are demonstrated in this view. The anterior portion of the longitudinal fissure (black arrow) depicts clear non-fusion of the frontal lobes. The frontal horns and trigone of the lateral ventricular components of the monoventricle can be visualized at the level (black arrowheads). (C) White arrow demonstrating the dorsal, midline interhemispheric cyst

The presence of a monoventricle is a rare finding that may suggest the diagnosis of semilobar holoprosencephaly as the initial primary consideration. In the patient presented, it was indeed the first differential considered after the initial radiographic reading. The patient showed several characteristics that are typical of holoprosencephaly: a dominant monoventricle with rudimentary frontal and temporal horns, absent septum pellucidum, and agenesis of the corpus callosum (Figures 1, 3, 4). On further inspection, the absence of cerebral structure fusion ruled out the diagnosis of holoprosencephaly. Although the ventricular features presented by the patient are characteristic of holoprosencephaly, these are not a deciding factor in the diagnosis. In this patient, there is a clear longitudinal fissure delineating the frontal lobes (Figures 1a, 4b). The basal ganglia and thalamic bodies are distinct, showing no evidence of fusion (Figure 3). The absence of frontal lobe fusion helps to rule out the diagnosis of holoprosencephaly. In the presence of unfused cerebral structures, the ventricular features exhibited by this patient best supports the identification of callosal agenesis with an interhemispheric cyst, as described by Barkovich et, al. [11].

## Conclusions

Agenesis of the corpus callosum with an interhemispheric cyst and Dandy-Walker malformation are pathologies of the brain that can be well identified on imaging of the head and have a broad range of clinical outcomes. Such a co-occurrence has been documented in pediatric populations, but there is little literature describing the presentation of adult patients. The patient presented here demonstrated a dominant monoventricle with rudimentary frontal and temporal horns, corpus callosum agenesis, and an interhemispheric midline dorsal cyst. These features raised an initial diagnosis of holoprosencephaly, but the lack of fused cerebral structures casts doubt against this possibility. Instead, these findings represent the rare coexistence of corpus callosum agenesis and interhemispheric cyst, which are rarely reported in non-pediatric literature. The radiographic findings identified here may provide clues to understanding the unique pattern of this rare presentation, helping facilitate surgical and medical management in affected individuals.
